# REEP3 is a potential diagnostic and prognostic biomarker correlated with immune infiltration in pancreatic cancer

**DOI:** 10.1038/s41598-024-64720-2

**Published:** 2024-06-15

**Authors:** Guo-Hua Liu, Xiao-Yu Tan, Zhen-Yue Xu, Jia-Xing Li, Guo-Hui Zhong, Jing-Wei Zhai, Ming-Yi Li

**Affiliations:** 1 Department of Hepatobiliary and Pancreatic Surgery, Affiliated Hospital, Guangdong Medical University, Zhanjiang, 524000 Guangdong China; 2 Institute of Surgery, Jinan University, Guangzhou, 510630 Guangdong China

**Keywords:** REEP3, Pancreatic cancer, Biomarker, Diagnostic, Immune infiltration, Cancer, Computational biology and bioinformatics, Medical research

## Abstract

Receptor Expression-Enhancing Protein 3 (REEP3) serves as a pivotal enzyme crucial for endoplasmic reticulum (ER) clearance during mitosis and is implicated in the advancement of diverse malignancies. Nonetheless, the biological role and mechanisms of REEP3 in pancreatic cancer patients, along with its interplay with immune infiltration, remain inadequately elucidated. In this study, we initially analyzed the differential expression of REEP3 between pancreatic cancer tissues and normal pancreas tissues using the Cancer Genome Atlas (TCGA), GTEx and Gene Expression Omnibus (GEO) databases. Subsequently, we utilized Kaplan–Meier analysis, Cox regression and ROC curve to determine the predictive value of REEP3 for the clinical outcomes of pancreatic cancer patients. Functional enrichment analyses, including Gene Ontology (GO), Kyoto Encyclopedia of Genes and Genomes (KEGG) and Gene Set Enrichment Analysis (GSEA), were conducted to explore the potential signaling pathways and biological functions associated with pancreatic cancer. Furthermore, we investigated the PPI network, miRNA, RBP and transcription factor interactions of REEP3 using databases such as GeneMania, STRING, StarBase, KnockTK, ENCODE, Jaspar and hTFtarget. Lastly, the “ssGSEA” algorithm and TIMER database were employed to investigate the correlation between REEP3 expression and immune infiltration as well as immune checkpoints. The expression of REEP3 in pancreatic cancer showed a significantly higher level compared to that in normal tissues. ROC curve analysis indicated that REEP3 holds substantial diagnostic potential for pancreatic cancer patients. Elevated REEP3 expression correlated with unfavorable outcomes in terms of both overall survival and relapse-free survival, establishing it as a notable adverse prognostic marker in pancreatic cancer. Moreover, both univariate and multivariate Cox regression analyses demonstrated that REEP3 maintained an independent association with overall survival. Functional enrichment analyses revealed pathways significantly linked to REEP3, including cytoplasmic translation, wound healing, viral processes, regulation of cellular component size and actin filament organization. Additionally, REEP3 expression displayed a significant positive correlation with CD8+ T cells, B cells, natural killer cells, dendritic cells and macrophages. REEP3 is a potential diagnostic, prognostic marker and immunotherapeutic target for pancreatic cancer.

## Introduction

Pancreatic cancer is characterized by its high invasiveness and malignancy. While it may not be the most prevalent form of cancer worldwide, it is among the deadliest tumors, exhibiting a poor prognosis and a global five-year survival rate of only 8%^1^. One of the clinical characteristics of pancreatic cancer is its heterogeneity, which can give rise to various subtypes and subgroups with unique molecular and cellular features, resulting in a wide range of clinical presentations^2,3^. The heterogeneity of pancreatic cancer presents challenges in its treatment. Main treatment options include surgery, chemotherapy, radiation therapy and targeted therapy^4^. Patients are often diagnosed at an advanced stage due to the lack of noticeable early symptoms, limiting the effectiveness of surgical resection and other conventional treatments. While some markers can assist in diagnosis, they may not provide adequate sensitivity and specificity^5,6^. Therefore, there is an urgent need to identify reliable biomarkers and molecular targets for the diagnosis and treatment of pancreatic cancer. By conducting in-depth research on the molecular mechanisms and biological characteristics of pancreatic cancer, we can enhance our understanding of prognostic factors and discover new therapeutic targets and strategies to enhance treatment outcomes and increase survival rates.

Receptor Expression Enhancing Protein 3 (REEP3) is a member of the REEP family, which includes REEP1, REEP2, REEP3, REEP4, REEP5 and REEP6. This protein family plays crucial roles in endoplasmic reticulum morphology and remodeling^7–9^, regulation of the microtubule cytoskeleton^10,11^ and the transport and expression of G protein-coupled receptors (GPCRs)^12,13^, among other important pathophysiological processes. REEP3 is extensively expressed in various tissues and cell types, characterized by an N-terminal cytoplasmic domain, a C-terminal cytoplasmic domain and four transmembrane domains (TMs)^7^. Initially discovered for its interaction with cell surface receptors, REEP3 plays a role in enhancing receptor expression and stability^12^. Research indicates that REEP3 is implicated in the regulation of endoplasmic reticulum remodeling and interacts with cytoskeletal proteins, influencing cell morphology and movement^11,14^. REEP3 is known to play a role in adipocyte generation and differentiation^15^. Recent studies have shown that REEP3 is significantly expressed in different types of tumors, with implications for tumor invasiveness and prognosis^16,17^. This suggests that REEP3 could potentially enhance the migratory and invasive abilities of tumor cells by influencing cell adhesion and cytoskeletal rearrangement. However, the specific involvement of REEP3 in pancreatic cancer remains unclear and requires further investigation.

The study initially examined the differential expression of the REEP3 gene in normal tissues and pancreatic cancer tissues using data from TCGA, GTEx and GEO databases. The aim was to assess its potential diagnostic and prognostic significance. Furthermore, the research analyzed the relationship between REEP3 expression and signaling pathways, immune infiltration levels and immune checkpoint genes to elucidate its biological functions and response to clinical treatments. Associations with clinical features were explored, along with investigating the potential role of REEP3 in tumor growth and progression. The findings offer insights into the underlying mechanisms of pancreatic cancer and present new targets and strategies for diagnosis and treatment.

## Materials and methods

### Data sources

The data for this study were gathered from the GEO, TCGA and GTEx databases. The GEO database (http://www.ncbi.nlm.nih.gov/geo) specifically hosts two separate datasets pertinent to pancreatic cancer: GSE62452 and GSE183795. Within the GSE62452 dataset, there are 69 cancer tissues and 61 normal controls, whereas the GSE183795 dataset comprises 139 cancer tissues and 105 normal controls. ^18^. The TCGA database (https://tcga-data.nci.nih.gov/tcga/) includes 178 pancreatic tissue samples and 4 normal pancreas tissue samples^19^. Furthermore, the GTEx database (https://gtexportal.org/home/) comprises 167 normal pancreas tissue samples^20^.

### Analysis of differential expression genes

Differential expression analysis of genes was conducted using the limma package in pancreatic cancer tissues compared to normal tissues to identify genes and pathways showing significant differences in expression. Genes meeting the screening criteria of *p* value < 0.05 and |LogFC|> 1 were considered differentially expressed. Among these, REEP3 emerged as a particularly intriguing candidate for further investigation due to its differential expression pattern.

### Functional enrichment analysis

We employed the Gene Set Enrichment Analysis (GSEA) tool and KEGG pathway analysis to conduct functional enrichment analysis on REEP3 and its associated genes, delving into their potential pathways and biological processes^21^. Additionally, we utilized GSEA analysis to ascertain the functional and pathway implications of differentially expressed genes.

### Constructing protein–protein interaction network

To gain deeper insights into the involvement of REEP3 in pancreatic cancer, we conducted screenings of genes and proteins interacting with REEP3 utilizing the GeneMania and STRING databases. Subsequently, we constructed gene–gene and protein–protein interaction networks. Additionally, we utilized the StarBase database to predict the miRNAs and RNA-binding proteins that potentially interact with REEP3. Moreover, employing databases such as StarBase, KnockTK, ENCODE, Jaspar and hTFtarget, we predicted the transcription factors responsible for regulating REEP3.

### Diagnostic and prognostic value analysis

We employed the “pROC” R package to generate ROC curves, with the area under the curve (AUC) serving as the evaluation criterion for diagnostic value: 0.50–0.60 = fail, 0.60–0.70 = poor, 0.70–0.80 = fair, 0.80–0.90 = good and 0.90–1 = excellent. Additionally, we utilized Kaplan–Meier curves and Cox regression models to assess the relationship between REEP3 expression and patient prognosis.

### Statistical analysis

To draw reliable conclusions, we conducted thorough statistical analyses to assess the significance of our findings. We employed appropriate statistical methods, including two-sample t-tests, analysis of variance (ANOVA) and chi-square tests, to rigorously analyze and interpret the results of our investigations into differential expression, correlation and prognostic analyses.

## Results

### REEP3 expression in pan carcinoma and pancreatic cancer

**Figure 1:**
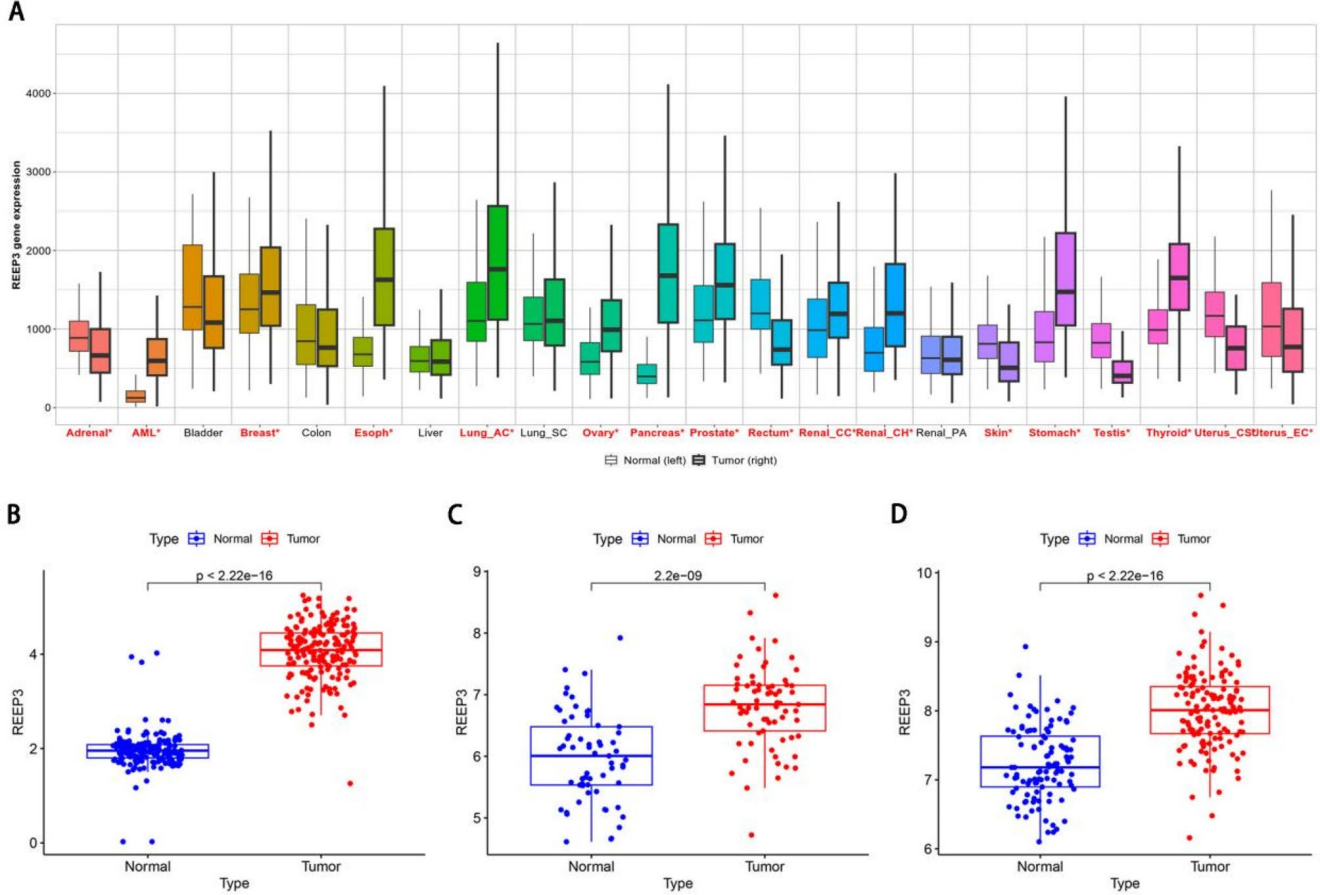
Expression analysis of REEP3 in the database. The expression pattern of REEP3 in types of cancer via Kaplan–Meier Plotter database. Significant differences by Mann–Whitney U test between cancer and normal tissues are marked with red*. No significant differences by Mann–Whitney U test between cancer and normal tissues are marked with black (**A**). Relative expression levels of REEP3 in TCGA and GTEx databases (N = 171, T = 178) (**B**). Expression of REEP3 in GSE62452 (N = 61, T = 69) (**C**). Expression of REEP3 in GSE183795 (N = 105, T = 139) (**D**).

Initially, we examined the expression of REEP3 across various normal and tumor tissues using the Kaplan–Meier Plotter database. Our analysis revealed a significant up-regulation of REEP3 in several tumors, including pancreatic cancer (Fig. 1A). Subsequently, we delved into the mRNA expression profiles of REEP3 within TCGA and GTEx cohorts. Our findings demonstrated a substantial up-regulation of REEP3 mRNA expression in pancreatic cancer compared to normal tissues (Fig. 1B). Moreover, this result was further validated by two additional GEO cohorts (Fig. 1C,D). In summary, the mRNA expression level of REEP3 in pancreatic cancer tissue was markedly elevated in comparison to that in normal pancreatic tissue.

### Potential diagnostic and prognostic value in pancreatic cancer

**Figure 2:**
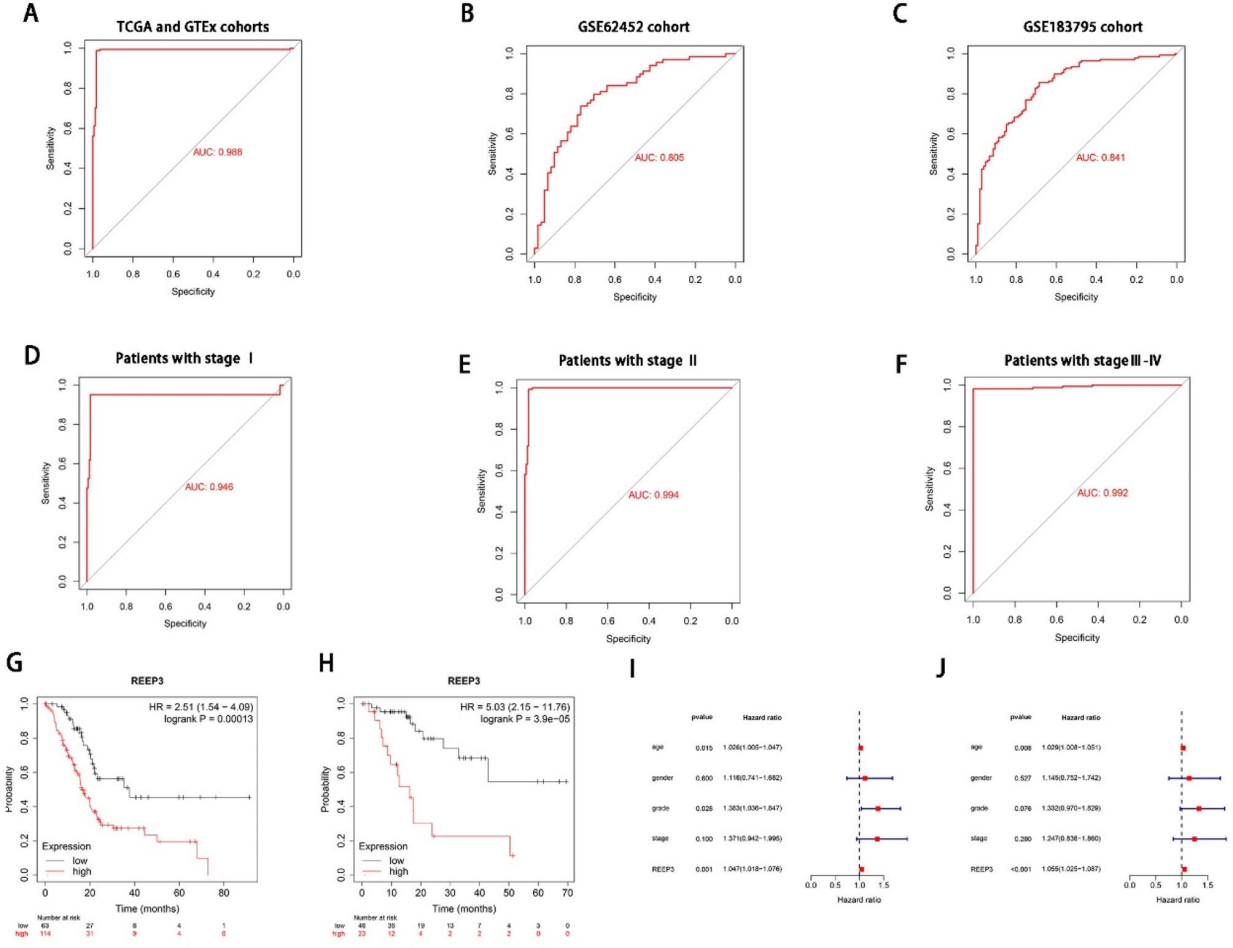
Potential clinical value of REEEP3 in pancreatic cancer patients. ROC curves of REEP3 expression in TCGA, GTEx and GEO cohorts (**A**–**C**). ROC curves of REEP3 expression in TCGA cohort with different pathological stages (**D**–**F**).The Kaplan–Meier curves about the correlation between REEP3 expression and overall survive of all patients (**G**).The Kaplan–Meier curves about the correlation between REEP3 expression and Relapse free survival of all patients (**H**). Univariate and multivariate Cox analyses of REEP3 and pathological characteristics (**I**,**J**).

Since the expression of REEP3 in pancreatic cancer tissue significantly deviates from that in normal tissue, there arises a speculation regarding REEP3’s potential diagnostic and prognostic significance in pancreatic cancer. Initially, we employed ROC curve analysis to assess the diagnostic efficacy of REEP3. Remarkably, the area under the curve (AUC) for REEP3 in the TCGA, GTEx, GSE62452 and GSE183795 cohorts was found to be 0.988, 0.805 and 0.841, respectively (Fig. 2A–C). Furthermore, an investigation into REEP3 expression across different stages within the TCGA cohort revealed notable AUC values for stages I, II, III and IV, namely 0.946, 0.994 and 0.992, respectively (Fig. 2D–F). Subsequently, we delved into the correlation between REEP3 expression and pancreatic cancer prognosis utilizing the Kaplan–Meier Plotter database. Intriguingly, the high REEP3 expression group exhibited significantly poorer overall survival (OS) and recurrence-free survival (RFS) prognosis in contrast to the low-REEP3 expression group (Fig. 2G,H). Subsequently, we conducted univariate and multivariate Cox regression analyses to assess the prognostic impact of REEP3 expression on pancreatic cancer patients. The outcomes underscored REEP3 as an independent predictor of overall survival among patients (Fig. 2I,J). In conclusion, these findings suggest that REEP3 expression could potentially serve as a biomarker for both the diagnosis and prognosis of pancreatic cancer patients.

### Identification of differential genes and functional enrichment analysis

**Figure 3:**
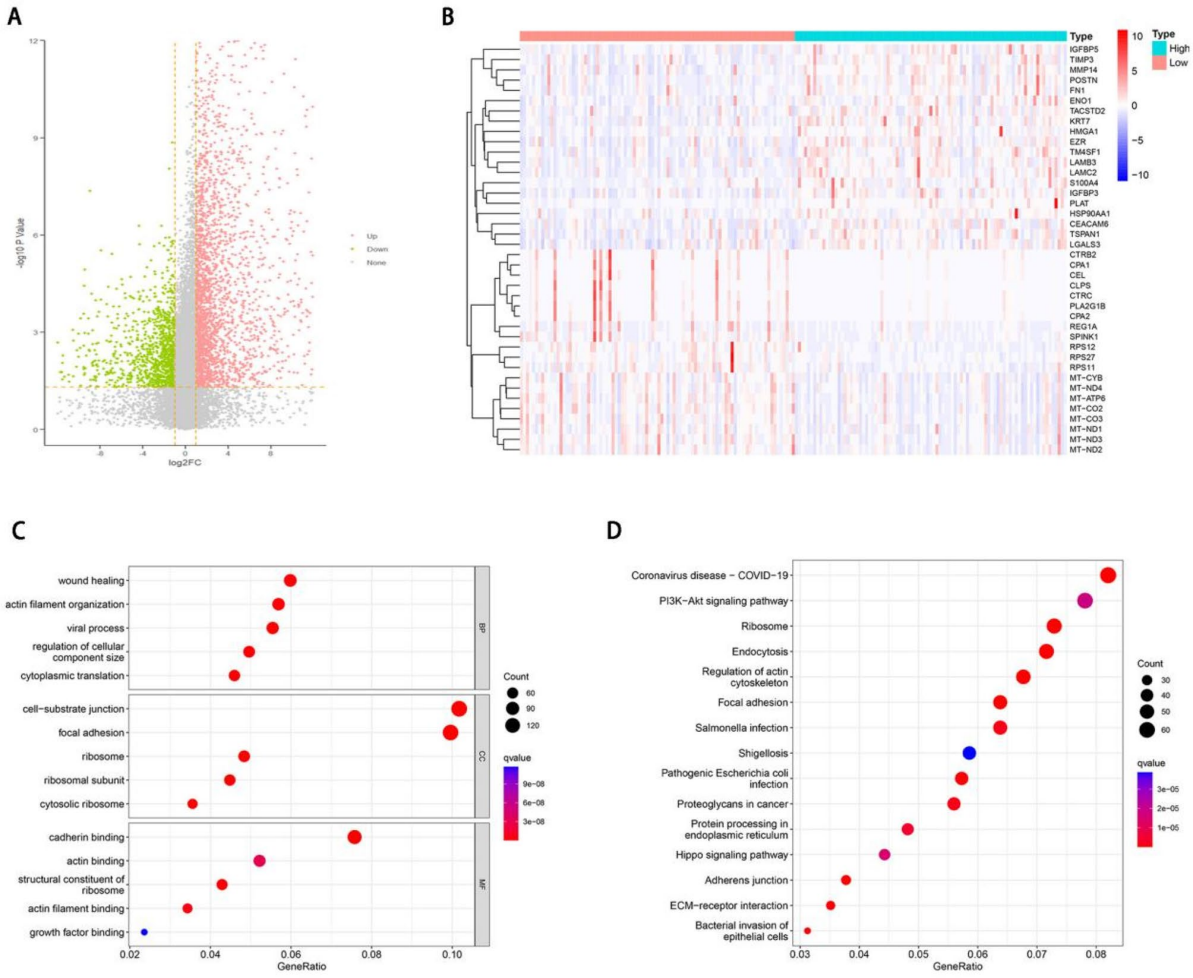
REEP3-related DEGs and functional enrichment analysis of REEP3 in pancreatic cancer using GO and KEGG. Volcano plot of DEGs in high REEP3 group in TCGA cohort compared with low REEP3 group (**A**). Heatmaps indicate the top 20 genes positively and negatively correlated with REEP3 in pancreatic cancer (**B**). Dot plot of the DEGs involving GO terms (**C**). Dot plot of the DEGs involving KEGG pathways (**D**).

**Figure 4:**
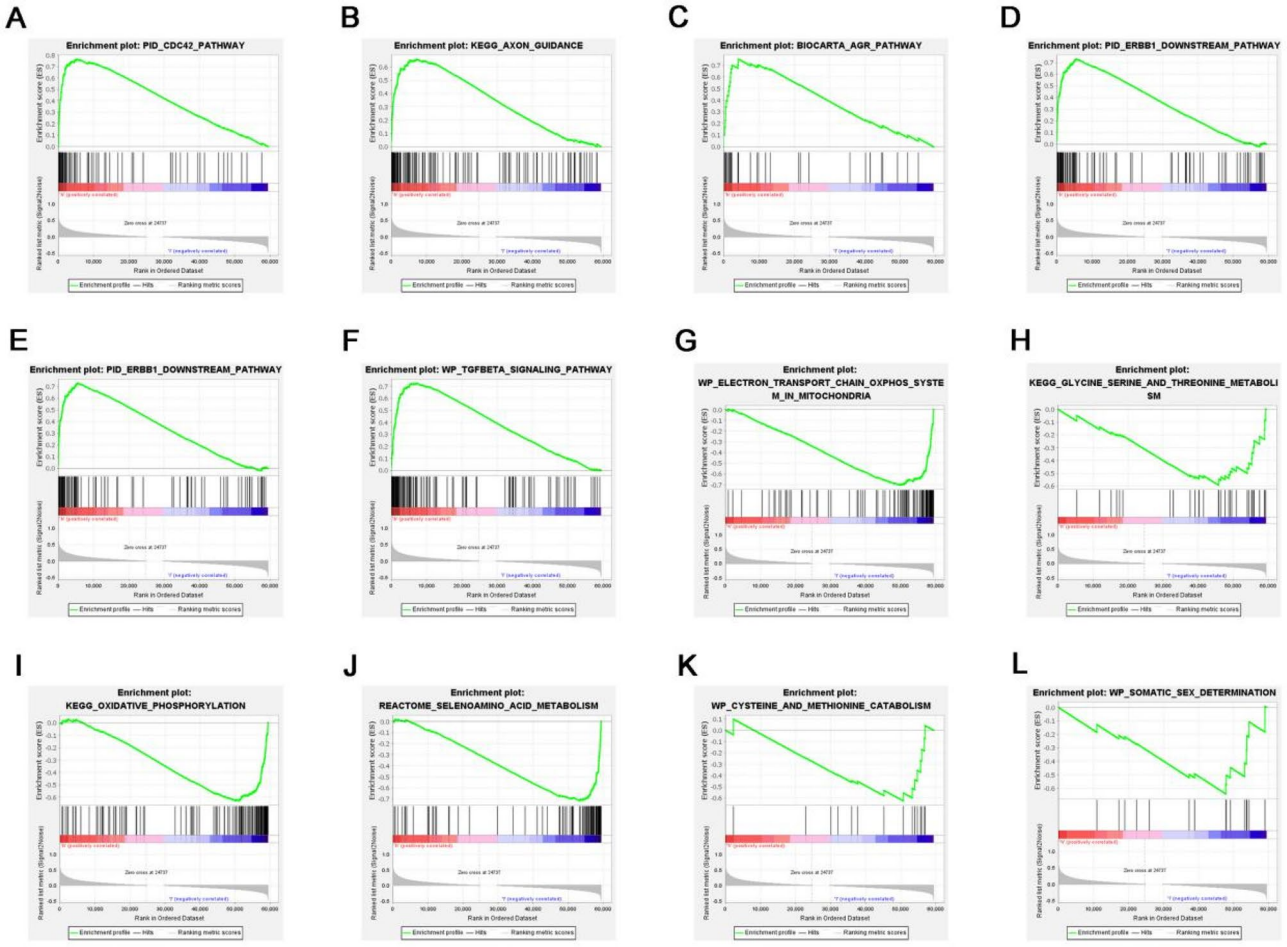
REEP3-related DEGs and functional enrichment analysis of REEP3 in pancreatic cancer using GSEA (**A**–**L**).

To elucidate the potential mechanism of REEP3 in pancreatic cancer, we stratified pancreatic cancer patients into two cohorts based on the median expression level of REEP3. We identified a total of 1475 genes with differential expression, comprising 1130 up-regulated genes and 345 down-regulated genes (Fig. 3A). The heat map of top 20 down‐regulated genes and top 20 up‐regulated genes (Fig. 3B). Subsequently, we conducted functional and pathway analyses of these differentially expressed genes using Gene Ontology (GO) and Kyoto Encyclopedia of Genes and Genomes (KEGG) databases. In terms of biological processes (BP), the enrichment analysis revealed that the differentially expressed genes were predominantly associated with wound healing, actin filament organization, viral processes, regulation of cellular component size and cytoplasmic translation. Concerning cellular components (CC), the differentially expressed genes were primarily enriched in cell-substrate junctions, focal adhesions, ribosomes, ribosomal subunits and cytosolic ribosomes. Moreover, molecular function (MF) analysis indicated enrichment in cadherin binding, actin binding, structural constituent of ribosome, actin filament binding and growth factor binding among the differentially expressed genes (Fig. 3C). KEGG pathway analysis demonstrated significant enrichment in pathways such as Coronavirus disease—COVID-19, Ribosome, Endocytosis, Regulation of actin cytoskeleton and Focal adhesion signaling pathways among the differentially expressed genes (Fig. 3D). Additionally, Gene Set Enrichment Analysis (GSEA) was conducted to identify specific signaling pathways associated with high and low REEP3 expression groups. The results revealed enrichment of pathways including TGFBETA_SIGNALING_PATHWAY, AR_PATHWAY, ERBB1_DOWNSTREAM_PATHWAY, AGR_PATHWAY, AXON_GUIDANCE and CDC42_PATHWAY in the high-expression group of REEP3. Conversely, pathways such as SOMATIC_SEX_DETERMINATION, CYSTEINE_AND_METHIONINE_CATABOLISM, SELENOAMINO_ACID_METABOLISM, OXIDATIVE_PHOSPHORYLATION, GLYCINE_SERINE_AND_THREONINE_METABOLISM and ELECTRON_TRANSPORT_CHAIN_OXPHOS_SYSTEM_IN_MITOCHONDRIA were predominantly enriched in the low-REEP3 expression group (Fig. 4).

### Constructing interaction network

**Figure 5:**
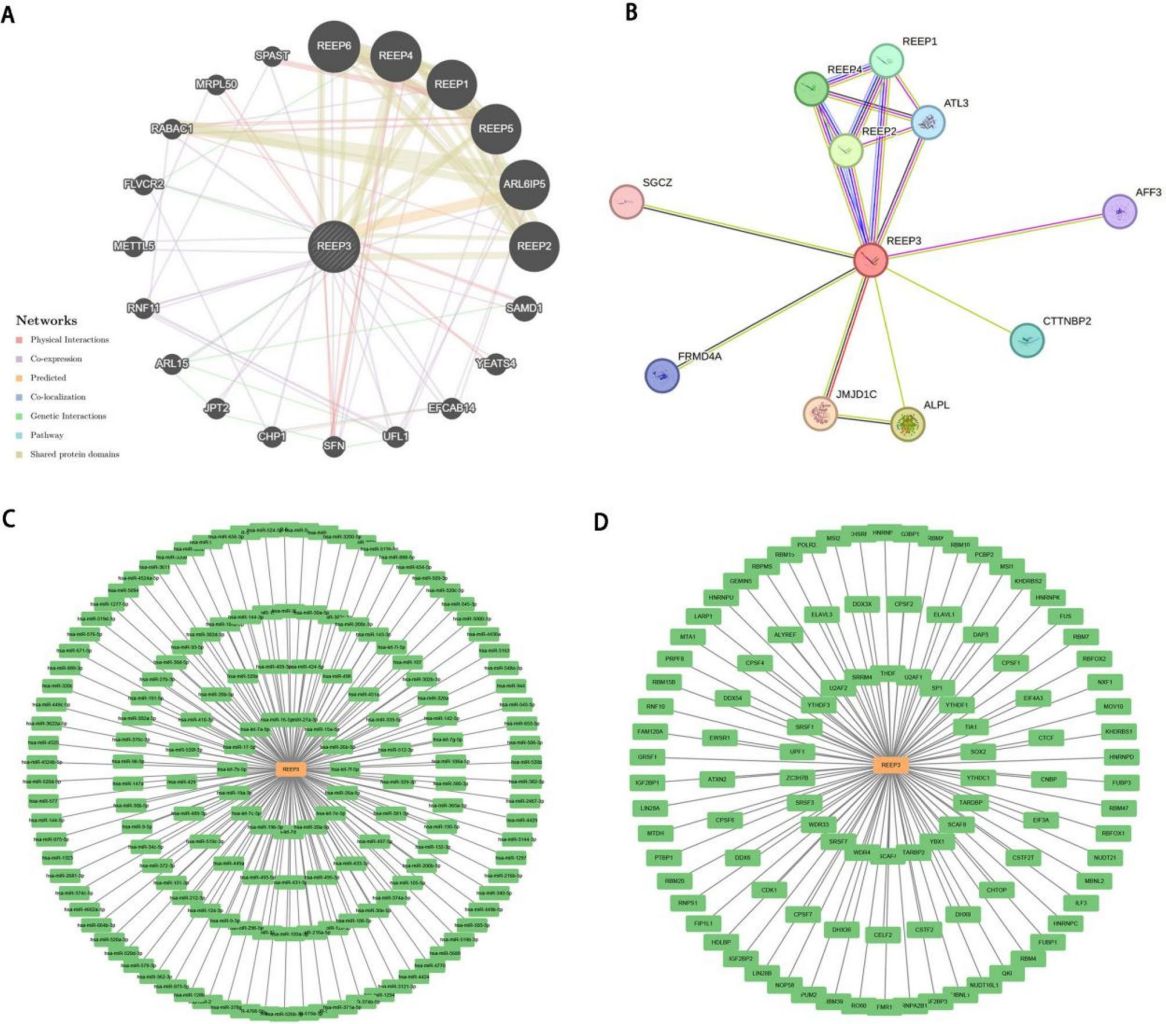
Construction of interaction network and related regulatory network. Gene interaction network of REEP3 based on the GeneMania database (**A**). PPI network of REEP3 based on the STRING database (**B**). The mRNA–miRNA network was constructed based on the starBase database (**C**). The mRNA–RBP network was constructed based on the starBase database (**D**).

We established a comprehensive gene interaction network involving REEP3, encompassing REEP1, REEP2, REEP4, REEP5, REEP6 and ARL6IP5, leveraging the GeneMania database (Fig. 5A). Additionally, we utilized the STRING online platform to explore the protein–protein interaction (PPI) network associated with REEP3, uncovering 35 edges and 11 nodes, which notably encompassed REEP1, REEP2 and REEP4 (Fig. 5B). Furthermore, our investigation extended to predicting the interaction network between REEP3’s miRNAs and RBPs, facilitated by the StarBase database (Fig. 5C,D).

### Prediction and identification of key transcription factors

**Figure 6:**
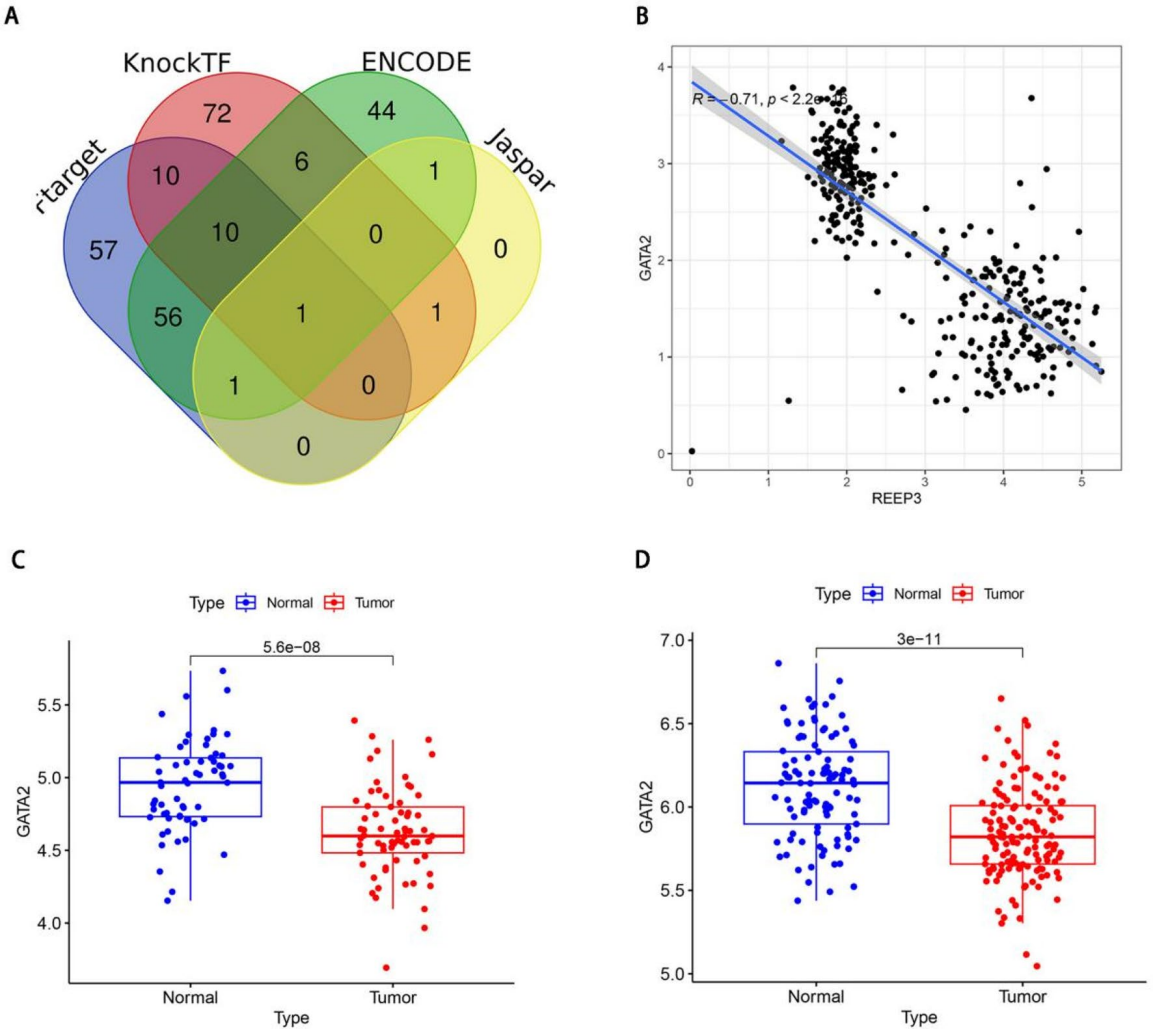
Identification of key transcription factors. Identification of GATA2 as a key transcription factor of REEP3 via KnockTK, ENCODE, Jaspar and hTFtarget databases (**A**). Correlation analysis of GATA2 and REEP3 in TCGA cohort (**B**). Expression of GATA2 in GSE62452 (N = 61, T = 69) (**C**). Expression of GATA2 in GSE183795 (N = 105, T = 139) (**D**).

We identified the transcription factor GATA2 associated with REEP3 through a comprehensive search across four online databases: KnockTK, ENCODE, Jaspar and hTFtarget (Fig. 6A). Subsequently, we conducted a detailed analysis of the correlation between REEP3 and GATA2. Our findings revealed a significant negative correlation between REEP3 and GATA2 expression levels (Fig. 6B). Furthermore, we examined the expression profile of GATA2 in two GEO datasets, uncovering a substantial down-regulation of GATA2 in tumor tissues compared to their normal counterparts (Fig. 6C,D). Notably, this expression pattern of GATA2 contrasts sharply with that of REEP3 in pancreatic cancer. These results strongly suggest a potential regulatory relationship between GATA2 and REEP3. However, the specific regulatory mechanisms remain unclear and warrant further investigation.

### Immune cell infiltration analysis

**Figure 7:**
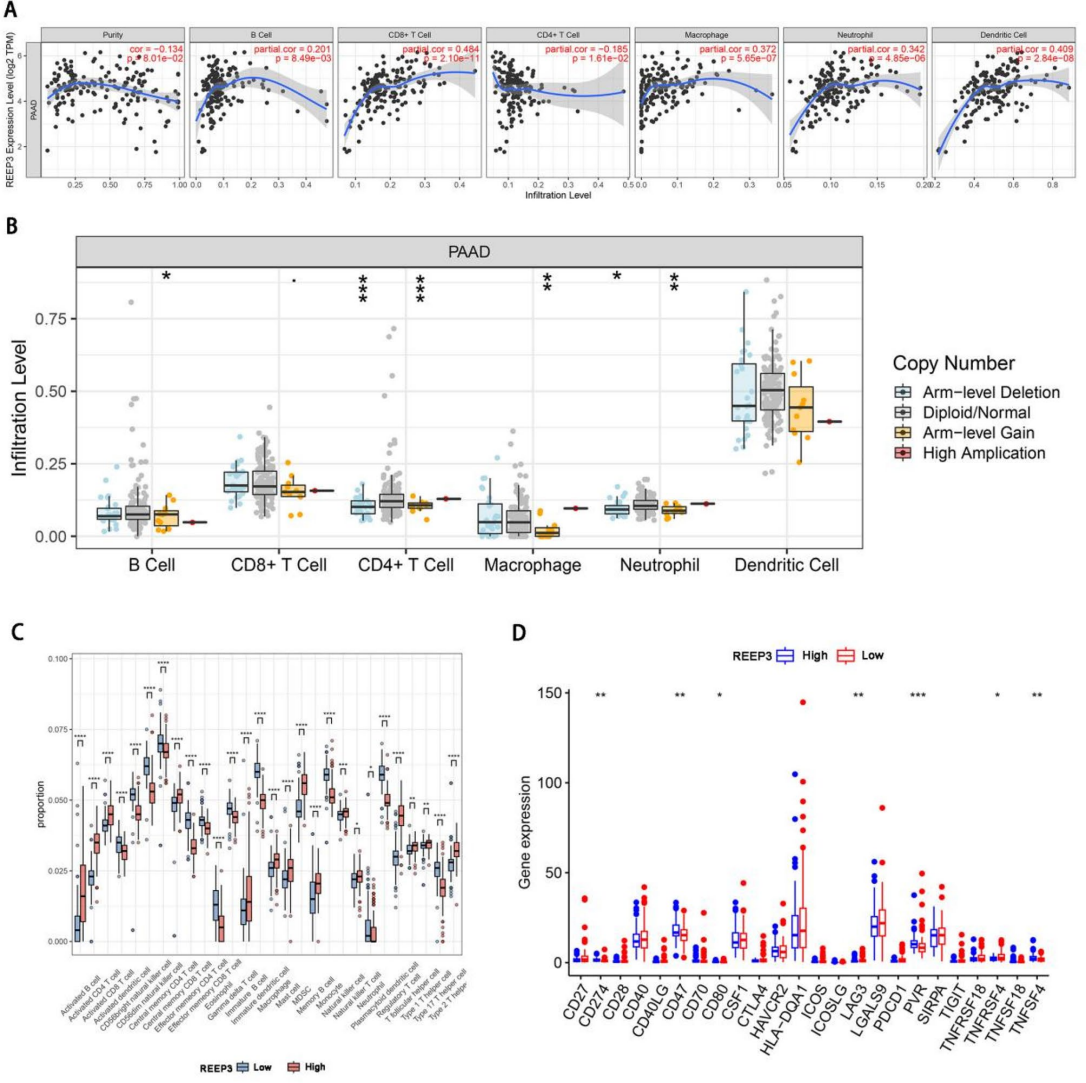
Correlations of REEP3 expression with immune infiltration in pancreatic cancer. Correlations of REEP3 with infiltrating levels of dendritic cells, macrophages, CD8+ T cells, neutrophils, B cells and CD4+ T cells in pancreatic cancer (**A**). Correlations of REEP3 mutation with immune infiltration (**B**). 28 subtypes for ssGSEA analysis of TCGA cohort in high and low REEP3 level patients (**C**). The expression of immune checkpoints in high and low REEP3 level patients (**D**).

We conducted an analysis to investigate the relationship between REEP3 expression and immune cell infiltration in pancreatic cancer using the TIMER database. Our findings revealed significant correlations between REEP3 expression and various immune cell types, including REEP3B cell (r = 0.201, *p* = 8.49E−02), CD8+ T cell (r = 0.484, *p* = 2.1E−11), macrophages (r = 0.372, *p* = 5.65E−07), neutrophils (r = 0.342, *p* = 4.85E−06) and dendritic cells (r = 0.409, *p* = 2.84E−08) (Fig. 7A). Additionally, REEP3 copy number variation (CNV) exhibited significant correlations with the infiltration levels of B cells, CD4+ T cells, macrophages and neutrophils (Fig. 7B). To delve deeper into the impact of REEP3 expression on the immune characteristics of pancreatic cancer, we utilized the “ssGSEA” algorithm to compare immune cell infiltration levels between the high-expression and low-expression groups of REEP3. Our analysis revealed that REEP3 high-expression was associated with increased infiltration of Activated B cells, Activated CD4 T cells, Activated CD8 T cells, Gamma delta T cells, Type 1 T helper cells, Type 2 T helper cells, Activated dendritic cells, CD56bright natural killer cells, CD56dim natural killer cells, Immature dendritic cells, Macrophages, Mast cells, Natural killer cells, Natural killer T cells, Plasmacytoid dendritic cells (*p* < 0.05). Conversely, infiltration levels of Central memory CD4 T cells, Central memory CD8 T cells, Effector memory CD4 T cells, Effector memory CD8 T cells, Immature B cells, Memory B cells, Regulatory T cells and T follicular helper cells were negatively correlated with REEP3 expression (*p* < 0.05) (Fig. 7C). Given the significance of immunotherapy in pancreatic cancer, we further investigated the correlation between REEP3 expression and immune checkpoint markers in pancreatic cancer patients. Our results indicated significant differences in the expression of immune checkpoint markers CD274, CD47, CD80, LAG3, PVR, TNFRSF18 and TNFSF4 between the high- expression REEP3 and low-REEP3 expression groups (Fig. 7D). These findings underscore the potential implications of REEP3 in modulating immune responses in pancreatic cancer and suggest its relevance in the context of immunotherapeutic interventions.

## Discussion

Pancreatic cancer is a highly lethal and aggressive malignancy, and its treatment remains challenging^22^. Traditional treatment modalities, including surgical resection, radiation therapy and chemotherapy, have yielded suboptimal results^23^. Immunotherapy, as a promising therapeutic approach, has made significant research progress in the field of pancreatic cancer treatment in recent years^24,25^. Immune checkpoint inhibitors, as important immunotherapeutic agents, activate T cells to exert cytotoxic effects on tumors by inhibiting programmed cell death protein 1 (PD-1), programmed death-ligand 1 (PD-L1) and cytotoxic T lymphocyte-associated antigen 4 (CTLA-4). Immune checkpoint inhibitors targeting the PD-1/PD-L1 pathway have shown remarkable therapeutic efficacy in multiple malignancies^26^, but their effectiveness in pancreatic cancer is limited^26^.

Our study revealed significant upregulation of REEP3 expression in a broad range of cancers, including pancreatic cancer. Through the analysis of multiple datasets including TCGA, GTEX, GEO and Kaplan–Meier Plotter databases, we observed a significant increase in REEP3 mRNA expression in pancreatic cancer patients, which was associated with unfavorable prognosis. This suggests that REEP3 may play an important role in the development of pancreatic cancer.

Given the differential expression of REEP3 in pancreatic cancer tissues compared to normal tissues, this gene has the potential to serve as a diagnostic and prognostic marker in pancreatic cancer. We further validated the diagnostic value of REEP3 by analyzing ROC curves and found high AUC values for REEP3 in the TCGA, GSE62452 and GSE183795 datasets. Additionally, the expression of REEP3 was found to be associated with the prognosis of pancreatic cancer at different stages.

To elucidate the potential mechanisms of REEP3 in pancreatic cancer, we identified differential expression genes and conducted functional enrichment analysis. Results revealed that these differential expression genes primarily function in biological processes such as wound healing and cell–matrix adhesion. Pathway enrichment analysis indicated enrichment of differential expression genes in signaling pathways such as focal adhesion. We also constructed a gene interaction network of REEP3 and analyzed its interactions with other genes, protein–protein interactions, as well as miRNA and RBP interactions. These analyses revealed associations between REEP3 and other genes and provided insight into potential regulatory mechanisms of REEP3 in pancreatic cancer. GATA2 belongs to the GATA family of transcription factors, promoting chromatin accessibility and interaction with other transcription factors and plays a crucial role in the development and differentiation of various cell types^27,28^. Research has revealed that high glucose levels can enhance the expression of polycomb protein Bmi1, leading to increased GATA2 levels and suppression of cell surface MICA/B expression, thereby facilitating immune evasion in pancreatic cancer cells^29^. Our study revealed a notable negative correlation between REEP3 and GATA2. Furthermore, disparate expression patterns of GATA2 and REEP3 were observed in two GEO datasets, suggesting a potential interaction between GATA2 and REEP3 in the progression of pancreatic cancer. Finally, we analyzed the correlation between REEP3 expression and immune cell infiltration in pancreatic cancer. Results showed a significant correlation between REEP3 expression and infiltration of B cells, CD8+ T cells, macrophages, neutrophils and dendritic cells. Furthermore, REEP3 copy number variation exhibited significant correlations with infiltration levels of B cells, CD4+ T cells, macrophages and neutrophils. Immune cell infiltration analysis further revealed differences in the infiltration levels of Plasmacytoid dendritic cells and Central memory CD4 T cells between high and low-REEP3 expression groups.

Based on our findings, REEP3 may serve as a potential therapeutic target in pancreatic cancer. Its association with adverse prognosis suggests that inhibiting or interfering with REEP3 function may help suppress the growth and metastasis of pancreatic cancer. Therefore, we performed drug sensitivity analysis to identify potential therapeutic drugs for pancreatic cancer. This could lead to the development of personalized treatment strategies for pancreatic cancer patients and improve their prognosis.

There are some limitations to this study. Firstly, it is based on retrospective analysis of clinical cases, which introduces the possibility of selection bias and incomplete information. Therefore, further prospective studies, multicenter studies and randomized controlled trials are necessary to validate our findings. Secondly, this study primarily relies on the analysis of gene expression and the correlation with clinical data. Functional validation experiments and animal model studies have yet to be conducted, underscoring the necessity for further experimental research to validate our conclusions. Future directions of research could contain the following aspects: Firstly, further investigation of the detailed mechanisms of REEP3 in pancreatic cancer development, particularly in pathways related to cell cycle regulation, cell proliferation and apoptosis. Secondly, further exploration of the role of REEP3 in pancreatic cancer metastasis and prognosis, as well as its potential value in personalized treatment. Lastly, exploring the interactions between REEP3 and other molecules through studying other related genes and signaling pathways, to obtain a deeper understanding of the mechanisms underlying the development of pancreatic cancer.

In summary, our study reveals the important role of REEP3 in pancreatic cancer and provides insights into its potential mechanisms. These findings contribute to a better understanding of the pathogenesis, diagnosis and prognosis evaluation of pancreatic cancer.

## Data Availability

Data are available from the corresponding author upon reasonable request.
